# PP67 From Insight To Foresight: Singapore’s Experience In Horizon Scanning Of Medical Technologies

**DOI:** 10.1017/S0266462324002381

**Published:** 2025-01-07

**Authors:** Zhen Long Ng, Hong Ju, Jaryl Ng, Swee Sung Soon, Kwong Ng

## Abstract

**Introduction:**

The Agency for Care Effectiveness (ACE) in Singapore has established a horizon scanning (HS) system to provide advance notice of new and emerging medical technologies (MedTechs) to inform service planning since 2020. To remain fit for purpose, HS needs to evolve over time to better serve its stakeholders. This abstract aims to share ACE’s early HS experience and future opportunities.

**Methods:**

To gather insights into ACE’s HS successes and potential gaps, a mixed-methods approach was used. A review of key HS activities was conducted, including different HS outputs produced, stakeholder engagements conducted, and dissemination strategies employed. Feedback from key stakeholders was gathered to understand the potential utility of our outputs. Lastly, potential gaps and opportunities were identified to inform future initiatives.

**Results:**

As of July 2023, 20 HS reports were developed, comprising three high-level overviews and 17 in-depth briefs. Output of briefs increased five-fold from 2021 (n=2) to 2023 (n=10). Efforts to drive impact included industry submission of pipeline MedTechs to supplement identification sources; report dissemination to over 100 policymakers and healthcare professionals for early service planning; and participation in network activities and conferences to share knowledge. Initial stakeholder feedback was positive, citing the reports’ relevance for clinical practice. The review recommended diversifying HS outputs to include environmental scan and trends reports, strengthening patient involvement and formal impact assessment through stakeholder surveys.

**Conclusions:**

ACE’s HS has made significant progress in achieving its intended objectives to enable service planning for new and emerging MedTechs. However, this remains an iterative process with new opportunities for growth. Future initiatives were identified, such as impact assessment, enhancing stakeholder engagement to include patients, and broadening outputs to meet stakeholder demands.

